# Effectiveness of Neuromodulation in Postoperative Pain Management Following Spine Surgery: A Systematic Review

**DOI:** 10.7759/cureus.83004

**Published:** 2025-04-25

**Authors:** Rahul Venna, Tomohiro Yamamoto, Adam Romman, Aristides P Koutrouvelis, Satoshi Yamamoto

**Affiliations:** 1 Anesthesiology, The University of Texas (UT) Medical Branch, Galveston, USA; 2 Medicine, Gunma University School of Medicine, Maebashi, JPN

**Keywords:** analgesia, neuromodulation techniques, neurosurgery, post operative pain management, spinal cord stimulation (scs), spinal surgery, transcranial direct current stimulation (tdcs), transcutaneous electrical nerve stimulation (tens)

## Abstract

Postoperative analgesia following spinal surgical interventions continues to present a formidable challenge, particularly due to the high prevalence of lumbar spine surgeries and the inherent risks linked to opioid-based analgesic strategies. Neuromodulation has emerged as a promising therapeutic modality for the management of pain associated with these procedures. A systematic review was undertaken to identify randomized controlled trials (RCTs) that investigate the effectiveness of neuromodulation techniques in patients undergoing lumbar spinal surgeries for postoperative pain relief. A comprehensive search of online databases was conducted following Preferred Reporting Items for Systematic Reviews and Meta-Analyses (PRISMA) guidelines. Randomized controlled trials investigating neuromodulation for postoperative pain relief following lumbar spinal surgery were included. Primary outcomes studied were opioid consumption and pain intensity scores. Through a systematic review, a limited selection of studies exploring neuromodulation techniques in patients undergoing lumbar spinal surgeries for postoperative pain relief was identified. Existing literature suggests potential benefits of transcranial direct current stimulation (tDCS) and transcutaneous electrical nerve stimulation (TENS) in reducing opioid consumption and alleviating postoperative pain. In individual randomized control trials within the review, TENS resulted in a 62% reduction in opioid use compared to sham, while motor/prefrontal tDCS had mixed results across trials. Findings from the studies suggest non-invasive neuromodulation techniques may potentially reduce postoperative opioid requirements after lumbar spine surgery. The smaller pool of available studies highlights a significant gap in research on neuromodulation for postoperative pain, especially for the lumbar surgery population. Further high-quality, large-scale randomized controlled trials are needed to investigate their functions within clinical practice.

## Introduction and background

Managing postoperative pain after spinal surgery is a significant challenge. The difficulty associated with the management of postoperative pain following spinal surgical interventions is paramount, particularly considering the increasing frequency of lumbar spine surgery coinciding with the growing prevalence of low back pain within the United States [1]. Several multimodal therapeutic approaches have been investigated and analyzed over the years to mitigate this concern [2]. Nearly 40% of those who have spinal surgery develop failed back surgery syndrome (FBSS), defined by lingering pain after undergoing spinal surgery [3]. Furthermore, the use of opioids for alleviating pain in the postoperative phase has considerable risks, especially within the framework of the persisting opioid epidemic [4]. 

Given these hurdles, neuromodulation has come forth as a treatment modality for handling pain [5]. Neuromodulation uses electrical, chemical, or magnetic stimuli aimed at altering neural activity and affecting pain pathway signaling. This framework utilizes the notion of neuroplasticity, recognized as the brain's capability to reshape itself by forming new neural links in response to various stimuli [6]. By concentrating on specific neural circuits implicated in pain perception, neuromodulation can modify pain signals and offer relief to patients [7]. A variety of neuromodulation techniques have garnered attention for their prospective utility in managing postoperative pain following spinal surgery. One such modality, known as spinal cord stimulation (SCS), involves the embedding of electrodes along the spinal cord to send electrical impulses that can obstruct pain signals from getting to the brain [8]. Additionally, SCS has demonstrated efficacy in diminishing pain intensity and enhancing the quality of life for patients afflicted with chronic pain conditions, including those suffering from FBSS [9]. 

Transcutaneous electrical nerve stimulation (TENS) represents another modality of neuromodulation and utilizes electrical currents applied to the skin to engage nerve fibers and lessen pain awareness [10]. Noted for being portable and non-invasive, TENS has found extensive application in addressing different pain pathologies. Neuromodulation strategies, including transcranial direct current stimulation (tDCS) and repetitive transcranial magnetic stimulation (rTMS), target the areas of the brain's cortex that are connected to the sensation of pain [11]. Of note, tDCS administers a low-intensity electrical current to the scalp, whereas rTMS employs magnetic pulses to stimulate designated brain areas. Both techniques have been subjected to investigation regarding their analgesic properties and have shown promise in modulating pain pathways and decreasing pain intensity. 

This systematic review investigates the success of neuromodulation techniques versus conventional pain relief options after spinal surgery. By evaluating parameters such as opioid consumption and pain intensity scores, this review aspires to quantify the disparities in pain relief afforded by neuromodulation techniques while ascertaining their potential benefits over standard pain management approaches. The results of this review could shed light on the mechanisms of neuromodulation in controlling pain post-surgery and also contribute to crafting enhanced and safer strategies for managing pain in those experiencing spinal surgery. 

## Review

Methodology

This systematic review complied with the Preferred Reporting Items for Systematic Reviews and Meta-Analyses (PRISMA) standards. A comprehensive search was conducted across PubMed, ScienceDirect, MEDLINE, Google Scholar, and the Cochrane Library with no publication date restriction. The team conducted an initial search strategy using broad keywords such as "spinal surgery," "postoperative pain," "pain scale," and "neuromodulation." To ensure comprehensive coverage, reference lists of the studies identified in the search were manually reviewed to verify that no pertinent articles were overlooked.

Studies were included only if they were randomized controlled trials (RCTs) involving adults undergoing lumbar spinal surgery, including lumbar fusion, decompression, or laminectomy procedures. The studies had to involve the use of a neuromodulation technique as a postoperative intervention. Additionally, the inclusion criteria required that neuromodulation techniques be integrated into postoperative pain management, with a comparative group receiving either sham stimulation or a standardized postoperative pain management protocol. The key outcomes for assessment included opioid usage following surgery and pain severity, which were evaluated using the visual analog scale (VAS) and the Numerical Rating Scale (NRS).

Studies were excluded if they were categorized as case reports, editorials, or narrative reviews. Studies were excluded if they did not assess neuromodulation for postoperative pain in lumbar surgery (focused on cervical or thoracic procedures). Studies were excluded if they focused exclusively on chronic pain management. If the studies did not measure postoperative pain using a pain scale such as VAS and NRS. Furthermore, studies were also excluded if they primarily focused on pharmacological interventions, surgical interventions, investigated cervical spine operations, utilized neuraxial anesthesia or peripheral nerve blocks, or focused on physical therapy as the primary intervention.

Two independent reviewers (RV and TY) evaluated all articles for inclusion and exclusion based on their relevance to the study and the criteria mentioned above. Any discrepancies were resolved through mutual agreement. We registered the review in March 2025 with the International Platform of Registered Systematic Review and Meta-Analysis Protocols (INPLASY) with registration number INPLASY202530069. 

We evaluated each study's methodological rigor using the Cochrane Risk of Bias 2 (RoB 2) tool. The elements evaluated by the tool include randomization techniques, allocation concealment, blinding for participants and outcome reviewers, completeness of the data, and selective reporting. Furthermore, for these RCTs, the Jadad score was used to assess the internal validity of the studies. The Jadad scoring system ranges from 0 to 5, with 5 indicating the highest methodological thoroughness. Each study underwent a risk-of-bias evaluation to ensure transparency and reliability of the studies.

Results

Study Identification and Inclusion

Out of the initial 153 records identified, 149 were subsequently excluded based on the criteria delineated previously. The excluded studies focused on cervical or thoracic surgery, did not investigate postoperative pain, and did not use an appropriate pain assessment scale. We further evaluated whether they focused exclusively on pharmacological interventions, surgical interventions without the inclusion of neuromodulation, investigated cervical spine surgeries, neuraxial anesthesia, peripheral nerve blocks, or considered physical therapy as the primary modality of intervention. The records were also screened to exclude narrative reviews, case reports, or editorials.

**Figure 1:**
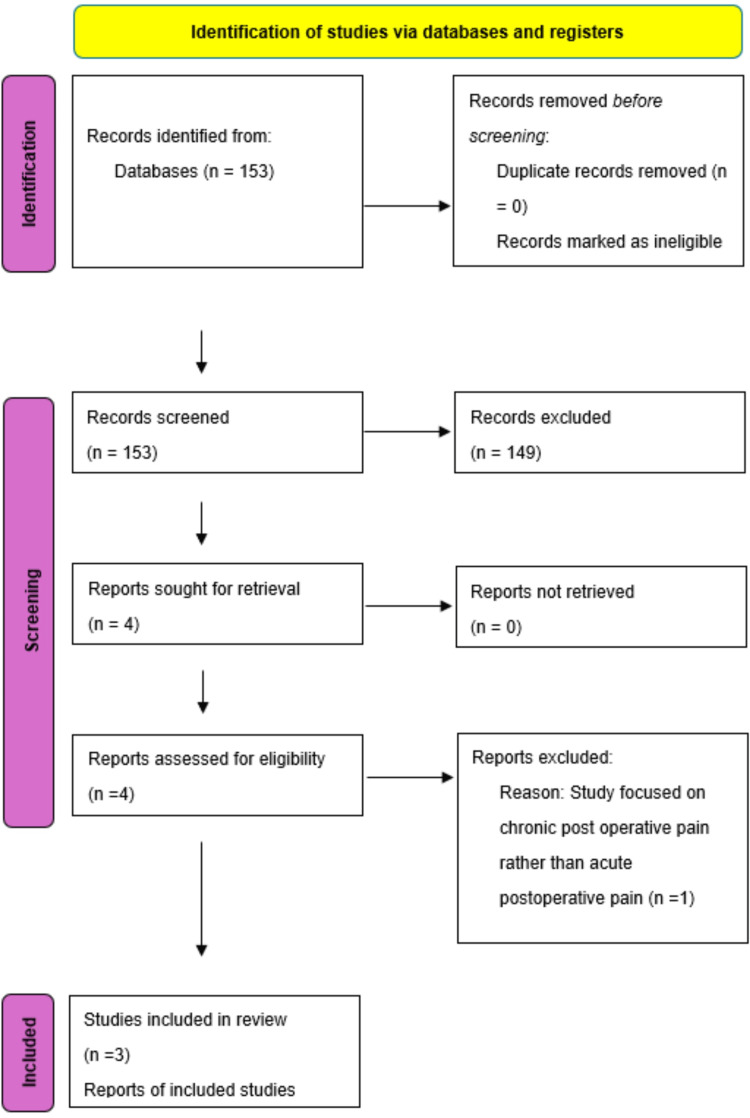
PRISMA flow diagram. PRIMSA (Preferred Reporting Items for Systematic Reviews and Meta-Analyses) for study selection.

After screening, the remaining four records underwent a comprehensive screening process. One of these four articles screened for eligibility was excluded as it incorporated neuromodulation for chronic rather than acute postoperative pain. Consequently, three articles remained for further analysis (Figure 1).

A total of 124 patients were included in the three studies and underwent major lumbar spinal surgery. Two studies assessed the impact of tDCS, while another examined TENS. The follow-up durations ranged from 24 hours to hospital discharge. The adverse effects were minimal. None of the studies used crossover designs (Table 1).

**Table 1:** Study characteristics. This review includes three randomized controlled trials by Dubois et al., Glaser et al., and Unterrainer et al. [12-14], conducted in Belgium, the United States, and Austria, and examines the effects of non-invasive neuromodulation techniques on postoperative pain following lumbar spine surgery. Funding included institutional grants and departmental resources. A total of 124 patients were included. All the patients included in the studies had major surgery on the lumbar spine. Two studies assessed the impact of transcranial direct current stimulation (tDCS), while another examined transcutaneous electrical nerve stimulation (TENS). Follow-up durations ranged from 24 hours to hospital discharge. Adverse effects were only seen in a study by Glaser et al. and included tingling, burning, itching in 26% of study participants at its highest with diminishing intensity across sessions [13]. There were no adverse effects reported in other two studies. None of the studies used crossover designs.

Study name (Author et al., Year)	Country	Type of study	Surgery subjects	Treatment level	Frequency	Device parameters	Setting	Crossover study	Funding source	Adverse effects	Follow-up timepoints
Dubois et al. (2013): Postoperative Analgesic Effect of tDCS in Lumbar Surgery [12]	Belgium	Randomized, double-blind, placebo-controlled trial	Lumbar Spine Surgery	Dorsolateral Prefrontal Cortex (DLPFC)	Single Session Postoperatively	1 mA, 20 minutes	Recovery Room	No	FRSM, Fondation van Goethem-Brichant, UCL	No reported adverse effects	PCA usage + pain assessment at 24 and 48 hours
Glaser et al. (2016): Motor/Prefrontal tDCS After Lumbar Surgery [13]	USA	Randomized, double-blind, sham-controlled pilot trial	Lumbar Spine Surgery	Motor Cortex + Prefrontal Cortex	4 Sessions Postoperatively	2 mA, 20 minutes/session	Hospital Post-Surgical Ward	No	NASS Grant	Mild (tingling, burning, itching)	PCA usage was tracked until discharge
Unterrainer et al. (2010): Postoperative and Preincisional TENS Reduce Opioid Requirement [14]	Austria	Randomized, single-blinded, sham-controlled trial	Major Spinal Surgery (Lumbar Interbody Fusion)	Dermatomal Level Near Incision Site	Pre-incisional + Postoperative Sessions	100 Hz, 10-20 mA, 0.25 ms pulse width	Hospital Post-Surgical Ward	No	Departmental Sources	No reported adverse effects	PCA usage tracked for 24 hours

The three studies evaluated electrical stimulation for postoperative pain relief in lumbar spine surgery. The sample sizes were 27, 59, and 38 patients, respectively. Mean ages ranged from 50.3 to 60.9 years. The gender distribution varied in both tDCS trials but was even in the TENS trial (Table 2).

**Table 2:** Study demographics. The three studies by Dubois et al. [12], Glaser et al. [13], and Unterrainer et al. [14] examined electrical stimulation for postoperative pain in lumbar spine surgery.

Study name	Total patients	Male patients	Female patients	Male (%)	Female (%)	Mean age (years)
Dubois et al. (2013) [12]	59	27	32	45.8	54.2	50.3
Glaser et al. (2016) [13]	27	10	17	37	63	59.4
Unterrainer et al. (2010) [14]	38	19	19	50	50	60.9

All three studies were considered *high quality*, with two earning a perfect 5/5 on the Jadad Scale and one scoring 4/5. These studies exhibited methodological rigor with appropriate randomization, adequate blinding, and proper documentation of participant withdrawals (Table 3).

**Table 3:** Jadad quality assessment. All studies were checked for randomization, blinding, and participant withdrawals. The Jadad scores were used to assess the methodological quality of the randomized controlled trials reviewed. Randomization, blinding, and withdrawals/dropouts were all thoroughly evaluated. Thus, three studies were evaluated as high quality, with two receiving a 5/5 on the Jadad score and one receiving a 4/5.

Study name	Randomization	Blinding	Withdrawals/Dropouts	Jadad score
Dubois et al. (2013) [12]	2	2	1	5
Glaser et al. (2016) [13]	2	2	1	5
Unterrainer et al. (2010) [14]	2	1	1	4

As per the Cochrane risk-of-bias assessment tool, two of the investigations exhibited a low risk of bias, while one presented a moderate risk of bias (Table 4). Table 5 summarizes and provides an overview of the three empirical investigations on tDCS and TENS.

**Table 4:** Cochrane risk-of-bias assessment. The studies were evaluated using the Cochrane risk-of-bias tool. Factors assessed include random sequence generation, allocation concealment, blinding of participants and personnel, blinding of outcome assessment, incomplete outcome data, selective reporting, and other sources of bias. Two of the studies were considered to have a low risk of bias, with one being a moderate risk of bias. Of note, Unterrainer et al. had a moderate risk of bias because the study was single-blinded. While the patients were blinded [14], the administrators of the TENS intervention were aware of the allocation of treatment. This makes this a moderate risk of performance bias. TENS, transcutaneous electrical nerve stimulation

Study name	Random sequence generation	Allocation concealment	Blinding of participants and personnel	Blinding of outcome assessment	Incomplete outcome data	Selective reporting	Other sources of bias	Overall risk of bias
Dubois et al. (2013) [12]	Low risk	Low risk	Low risk	Low risk	Low risk	Low risk	Low risk	Low risk
Glaser et al. (2016) [13]	Low risk	Low risk	Low risk	Low risk	Low risk	Low risk	Low risk	Low risk
Unterrainer et al. (2010) [14]	Low risk	Low risk	Moderate risk	Low risk	Low risk	Low risk	Low risk	Moderate risk

**Table 5:** Experimental outcomes. This table summarizes three studies examining the effects of transcranial direct current stimulation (tDCS) or transcutaneous electrical nerve stimulation (TENS) on outcomes. The results shown in these articles show that motor/prefrontal tDCS and TENS may help reduce opioid usage. However, anodal and cathodal tDCS over the dorsolateral prefrontal cortex (DLPFC) showed no significant effect.

Study name	Experimental group(s)	Control group	Primary outcome
Dubois et al. (2013) [12]	Anodal tDCS (DLPFC), Cathodal tDCS (DLPFC)	Sham tDCS	No significant reduction in PCA morphine usage. No significant difference in VAS pain scores at rest or movement.
Glaser et al. (2016) [13]	Motor/Prefrontal tDCS (2 mA, 20 minutes, four sessions)	Sham tDCS	23% reduction in PCA opioid usage. Significant 31% pain reduction but no significant difference in average or worst pain scores.
Unterrainer et al. (2010) [14]	Pre-incisional + postoperative TENS, postoperative TENS	Sham TENS	62% opioid reduction in pre-incisional + postoperative TENS group vs. sham. Significant pain reduction

Discussion

The scope of clinical investigations using neuromodulation for postoperative pain remains limited. tDCS uses a direct current to the skull to impact neuronal operations in the cerebral cortex. Its attributes of simplicity, cost-effectiveness, and portability are highly regarded [15]. The efficacy of tDCS is believed to arise from its modulation of the primary motor cortex and the prefrontal regions, both of which are integral to the perception and modulation of pain [16]. Two studies have examined the analgesic effects of tDCS in the context of pain following spinal surgery. Glaser et al. reported a significant drop in postoperative opioid consumption tied to tDCS [13], whereas Dubois et al. did not find significant changes in opioid use or pain scores postoperatively [12]. Neither investigation revealed a significant inter-group disparity in postoperative pain ratings. While tDCS may facilitate a reduction in opioid utilization, it may not have a significant effect on postoperative pain relief.

TENS involves applying low-voltage electrical currents through surface electrodes to help ease pain, working by activating inhibitory pathways and prompting the release of endogenous opioids within the body [17]. A solitary study scrutinized the efficacy of TENS in the management of postoperative pain following spinal surgery. Unterrainer et al. focused on individuals experiencing postoperative pain after lumbar spinal surgery [14]. The study demonstrated that patients receiving pre-incisional and postoperative TENS had significantly lower opioid consumption compared to sham and postoperative-only TENS groups [14]. Limitations of the study regarding the randomization process were raised due to inadequacies in the assignment of patients to groups, thereby introducing a potential risk of selection bias stemming from possible patients' ability to predict their assigned group. The single-blinded structure of the study increased the likelihood of performance bias since the investigators were informed of group distributions, potentially shaping the study results.

Within these studies, there are observed differences in analgesic outcomes across studies, which could be due to the heterogeneity in stimulation protocols. Glaser et al. utilized 2 mA tDCS over multiple sessions [13], targeting both motor and prefrontal regions, while Dubois et al. applied a single 1 mA session over the DLPFC alone [12]. This could play a role in why Glaser et al. yielded pain control. Unterrainer et al. used pre-incisional timing of TENS and higher stimulation intensities (10-20 mA), which may have played a role in its opioid sparing effect [14]. This highlights both a gap in research and a need for standardization of protocols to better understand the effect of neuromodulation techniques.

There is an absence of clinical investigations on spinal cord stimulation (SCS) or repetitive transcranial magnetic stimulation (rTMS) and their effects on acute postoperative pain. While the prevalence of SCS for chronic pain varies from 1.1% to 4.3%, its use for acute postoperative pain has not been established. No human studies have been performed, but animal studies indicate the potential of SCS for the management of postoperative pain following spinal surgery [18] and its applicability for acute pain relief [19].

While both tDCS and TENS are posited to be advantageous, the intricate mechanisms through which these modalities modulate pain perception continue to be unclear and are being investigated. For example, tDCS is thought to affect cortical excitability and synaptic plasticity, thereby potentially modifying the neural networks engaged in pain processing. tDCS modulates cortical excitability by delivering a low-intensity direct current to the brain, which can alter neuronal activity in targeted areas. This modulation can affect both cortical and subcortical regions involved in pain processing, potentially enhancing descending inhibitory mechanisms that reduce pain perception [20,21]. The modulation of the primary motor cortex may influence descending pain pathways, resulting in altered pain perception [22]. TENS is postulated to activate the endogenous opioid system and modulate pain via the gate control theory, wherein non-painful stimuli can overshadow and diminish the perception of pain [23]. TENS works by stimulating peripheral sensory nerves, which serve as the non-painful input. This leads to inhibition of pain signals at the spinal cord level and the activation of descending inhibitory pathways originating in the brainstem [24,25].

The individualized responses to neuromodulation, including tDCS and TENS, reveal the nuanced aspect of pain mechanisms and the need for specialized pain management techniques [26]. Variables, including electrode positioning, stimulation parameters, and unique neurophysiological characteristics, can profoundly affect the efficacy of these interventions.

This systematic review has a few limitations. First, the number of included studies is three, which limits the generalizability of the findings. Furthermore, as there is heterogeneity in study designs and outcomes, it is very difficult to make direct statistical comparisons. Finally, SCS and rTMS, which are popular neuromodulation techniques, were not represented due to a lack of eligible RCTs.

Future investigations should prioritize the optimization of neuromodulation parameters while elucidating the long-term effects and underlying mechanisms of action associated with these techniques. Future research could systematically evaluate parameters such as intensity, number of sessions, and timing of surgery to further fill the gap in research. Conducting extensive, double-blind, randomized controlled studies is crucial to confirm the effectiveness and safety of neuromodulation techniques in managing postoperative pain. Furthermore, further research into SCS and rTMS in the acute postoperative phase of lumbar spinal surgery is needed, as there seems to be a clear gap in research on this subject. Finally, delving into the collaborative application of neuromodulation with alternative pain relief methods, such as medicinal treatments and physiotherapy, holds promise for boosting the success of postoperative pain management practices.

## Conclusions

Existing evidence indicates potential advantages of tDCS and TENS in mitigating opioid utilization following lumbar spinal surgery. TENS demonstrates a clear opioid-sparing effect, while tDCS trials are mixed in their efficacy for postoperative pain. These techniques show great potential, but current evidence is limited, and it should be interpreted cautiously. There remains a pressing need for more rigorous and comprehensive studies with clear standardization to delineate their roles within clinical settings. Continued advancements in neuromodulation research present significant promise for the formulation of more effective and individualized strategies for postoperative pain management.
